# Family Planning Knowledge, Attitudes, and Practices Among Male and Female Garment Factory Workers in Alexandria, Egypt

**DOI:** 10.1002/puh2.70174

**Published:** 2025-12-18

**Authors:** Noha el Khorazaty, Nahla Abdel‐Tawab

**Affiliations:** ^1^ Population Council Egypt Office Cairo Egypt

**Keywords:** Egypt, factory workers, family planning

## Abstract

Most evidence on factory workers’ family planning (FP) needs has primarily focused on female workers. This study examines knowledge, attitudes, and practices of male and female garment factory workers in Alexandria, Egypt, and identifies factors associated with the use of modern contraception among these two groups. Data were collected through face‐to‐face interviews with married male and female workers (406 and 393, respectively) in 14 factories whose owners agreed to participate in the study, whereas the sample of workers was composed of those whose supervisors approved of their participation in the interview. Bivariate analysis was used to measure differences in FP knowledge, attitudes, and behaviors. Factors associated with workers’ (or their spouse's) use of modern contraceptives were uncovered through separate logistic regression models. Although female workers could list more modern contraceptives than their male counterparts, female workers expressed more concerns and misconceptions about FP. The reported use of modern contraceptives was higher among male workers (or their spouses) than female workers. Factors predictive of modern contraceptive use among men were the number of children, the respondent's age, more positive attitudes, and knowledge of more FP methods, whereas among women, the only significant factor was having more children. The study highlights the need for gender‐responsive interventions to address the distinctive FP needs of male and female factory workers, on‐site provision of FP information and services in factory settings, and broader interventions to address inequitable gender norms.

## Graphical abstract

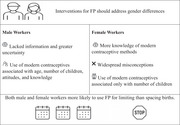

## 1 Introduction

Globally, the garment industry employs nearly 94 million workers, nearly 60% of whom are women [1]. Garment workers are predominantly in their reproductive years, many of whom have migrated from rural settings and traditional social contexts in search of better opportunities in urban centers [2, 3, 4, 5]. Evidence from countries with an expanding garment sector, such as Bangladesh, Cambodia, and Vietnam, has shown that workers in that sector have family planning (FP) and reproductive health (RH) needs that often go unmet. For example, many women working in Bangladesh's garment sector who were in the earlier half of their reproductive years had limited access to RH services and/or menstrual products [6]. In Cambodia, one study showed that three‐quarters of sexually active female garment factory workers were not using FP [7]. Another study showed that misconceptions about contraceptives were widespread among workers [8]. In Vietnam, a study revealed that female migrant workers who sought RH services faced barriers to accessing services, such as long work hours, lack of information, and high cost of services [9].

Most of the evidence on factory workers’ FP needs has been focused on females, although males constitute a significant proportion of the workforce in the garment sector. Even interventions that aimed at addressing workers’ FP needs did not distinguish between male and female workers when examining the effects of such interventions. A study that examined the effects of peer education along with referral of workers to private sector providers in Port Said, Egypt, showed modest improvements in workers’ FP knowledge, attitudes, and practices in intervention factories. For example, at endline, 43.7% of workers in intervention factories disagreed that FP methods can affect female fertility and may reflect negatively on “future pregnancies” (compared to 41.0% at baseline). Moreover, slightly more than one‐third of workers (38.9%) at endline knew three modern FP methods at endline (compared to 34.5% at baseline) [10]. However, the results were not disaggregated by sex, and hence differences in FP knowledge, attitudes, and practices between male and female workers before and after the intervention could not be ascertained.

Egypt, the focus of this article, has a large garment sector. As per the Egyptian Chamber of Apparel and Home Textile (ECAHT), the ready‐made garment and home furnishings sector employs more than 1.5 million formal workers and an additional 1–2 million informal/seasonal workers. The estimated ratio of female to male workers in the garment industry is 3:2, respectively. The sector contributes about 3% of the gross domestic product and covers 75%–80% of the local market's needs [11].

The present study examines FP knowledge, attitudes, and practices among married male and female garment factory workers in two industrial zones in Egypt and identifies factors associated with FP uptake among those workers and/or their spouses. The study is part of a larger project that aims at addressing FP needs of male and female factory workers in El‐Amereya industrial zone in Alexandria, Egypt, through on‐site provision of FP information and services. Understanding gender differences in FP knowledge, attitudes, and practices is crucial for designing gender‐responsive FP and RH (FP/RH) interventions and programs that ensure more equitable access and benefits for both men and women [12].

## 2 Methods

### 2.1 Study Setting

Egypt, with a population of 105 million, has an arduous population issue with an annual growth rate of 1.5% and a GDP growth rate of 3%, coupled with an escalating inflation rate currently at 19.7% [13]. The disparity between population and economic growth levels mounts pressure on the country's resources and underscores the need for FP.

Egypt has had a fairly successful FP program since the mid‐60s. According to Egypt's 2021 National Family Health Survey, the total fertility rate is estimated at 2.85; however, the unmet need for FP stands at 14%. Although basic knowledge of FP methods is high, with most ever‐married women aged 15–49 being aware of the three main modern methods (pill, IUD, and injectables), misinformation and misconceptions about FP methods are widespread among both married and unmarried men and women [14].

The public sector is the primary provider of FP services in Egypt, serving more than 62.5% of FP users [14]. Services are offered through more than 4000 public health units in rural and urban areas throughout Egypt. By law, all factories with more than 50 workers are required to have an infirmary that provides basic health services such as first aid. However, factory infirmaries do not provide FP services [15].

The factories selected for this study are in El‐Amereya Investment Zone and Borg El Arab Industrial Zone, both located in Alexandria governorate. El‐Amereya Investment Zone is 29 km south of Alexandria city and has 56 factories, of which 24 are garment factories [16]. Borg El Arab Industrial Zone is in Borg El‐Arab el Gadeeda city, 60 km southwest of Alexandria city, and has over 1270 factories that manufacture ready‐made garments, plastics, cosmetics, and other [17]. Both industrial areas employ workers of fairly similar backgrounds who live on the outskirts of Alexandria city [17]. Investors’ Association data indicate that the total number of formally employed workers in these zones is estimated at 35,000–45,000, more than half of whom (approximately 20,000–25,000) work in garment factories. Most workers commute to work by factory‐operated buses in return for a small fee that is deducted from their monthly salary.

### 2.2 Study Design

The study used a cross‐sectional survey design to assess FP knowledge, attitudes, and practices among a sample of married male and female workers in the above two industrial zones. The study protocol was reviewed and approved by the Population Council Internal Review Board on January 27, 2022.

### 2.3 Study Sample

A sample of 14 garment factories in El Amereya and Borg El Arab industrial zones were recruited for the project. The main selection criterion was that the factory had at least 30% female workforce. Of a total of 18 factories that were approached, 4 refused to participate in the project for fear that project activities might disrupt workers’ productivity or because managers did not see FP as a priority for their workers. The 14 selected factories had 6–21 production lines, depending on the factory size. The workforce in the factories ranged from 200 to 5000 workers, and the percentage of females ranged between 30% and 70%.

**FIGURE 1:**
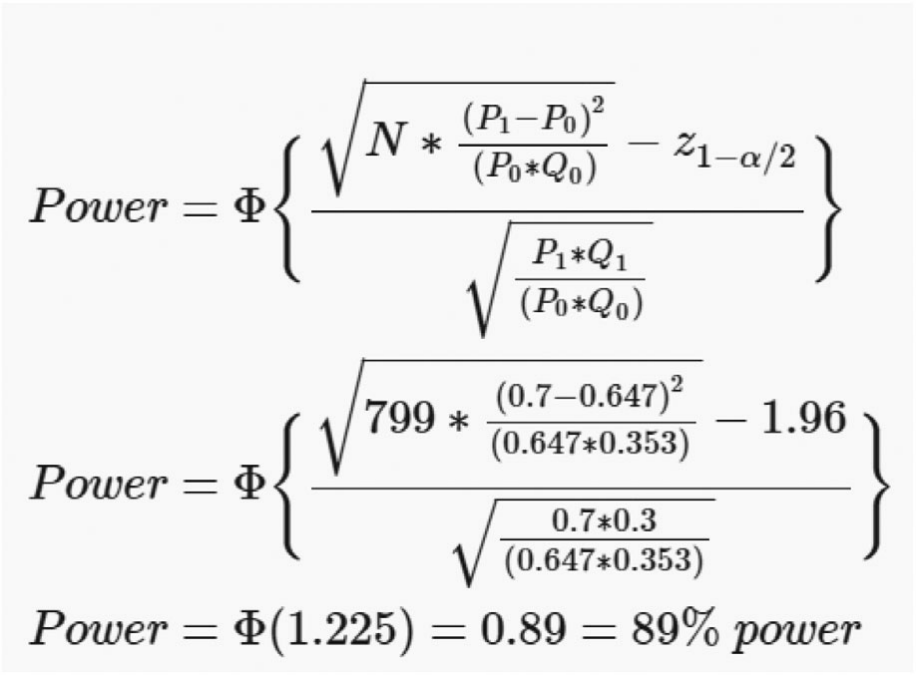
Calculations of post hoc study sample power.

The data presented in this article are a subset of data from a larger baseline survey that assessed male and female workers’ (married or unmarried) FP knowledge, attitudes, and practices prior to implementing the intervention. The current subset is limited to married male and female factory workers aged 18–49 who neither themselves nor their wives were pregnant at the time of data collection (i.e., who were eligible to be using FP). This dataset includes 799 married factory workers (406 males and 393 females). We focused this study on married workers, as cultural norms forbid pre‐/extramarital sexual relations, and hence, unmarried workers would be unlikely to report using FP methods. The sample power was estimated over 0.8 (calculated post hoc power = 0.89) (Figure 1).

### 2.4 Procedures for Data Collection

Before fielding the survey, the research team thoroughly explained over several meetings with factory managers the importance of workers’ participation in the baseline survey to identify their actual needs and to address an intervention that would address those needs. On each day of data collection, the study team informed the factory management of the number of workers who should be selected from each production line (e.g., cutting, sewing, and ironing). Managers and supervisors in each production line identified factory workers who fit the selection criteria, that is, age 18–49 and married, and released them from work to take part in the interview. Data collection was conducted through face‐to‐face interviews that were conducted by interviewers of the same gender as participants and were administered during lunch breaks. Each interview lasted for approximately 15 min and was conducted in a location that provided visual and auditory privacy.

Baseline data collection was conducted between April and August 2022. The collection of baseline data took longer than expected, as supervisors only allowed workers to take the interview during their lunch break so as not to disrupt production. On several occasions, data collection activities were put on hold because factories had purchase orders that had to be delivered at the time of data collection. Limited access to research participants is one of the challenges of conducting research in factory settings [10].

### 2.5 Variables and Measures

#### 2.5.1 Knowledge Indicators

The knowledge indicator used in this analysis was the number of modern contraceptives the respondent could name. Respondents were asked: *Can you name all FP methods that you know?* The data collectors were instructed to probe for all methods (modern or traditional) the respondent was aware of by asking: *Do you know any other methods?* Data collectors marked all the FP methods that were mentioned by respondents. Only modern methods available in the Egyptian market were listed on the questionnaire (listed in Figure 3). The knowledge score was computed by summing up the total number of modern hormonal or non‐hormonal methods named by the respondent.

#### 2.5.2 Attitudinal Indicators

**FIGURE 2:**
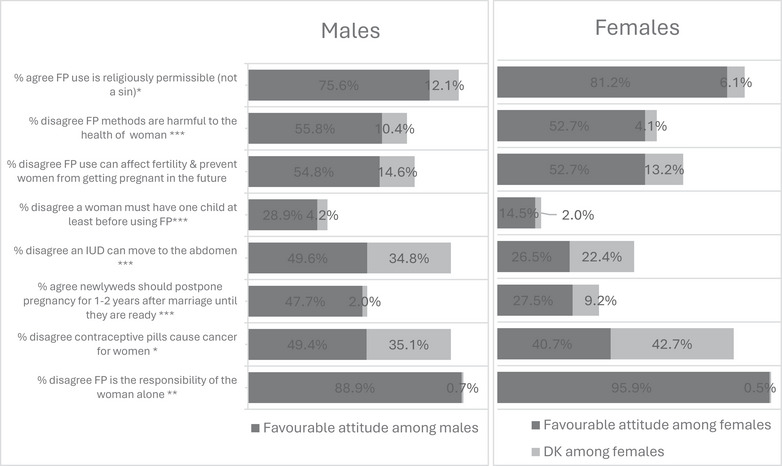
Percentage distribution of favorable attitudes towards FP among male and female workers (males = 405, females = 393). ^***^ *p* value ≤ 0.001; ^**^ *p* value ≤ 0.01; ^*^ *p* value ≤ 0.05. *Note:* One male refused to answer all attitudinal questions, thus excluded from the analysis presented in this figure.

The interviewer read to the respondent eight statements, some of which were negative statements conveying misinformation or misconceptions, whereas others were positive statements listed in Figure 2. Responses were captured on a 5‐point Likert scale ranging from strongly agree (4), agree (3), disagree (2), strongly disagree (1), and do not know (0).

For the regression model presented below (Tables 5 and 6), a combined indicator was constructed, grouping the four statements that pertained to misconceptions or misinformation about the effects of FP/contraceptives on women's health, “NegWomenHealth.” A higher score in NegWomenHealth reflects stronger unfavorable attitudes towards the effect of FP. After recoding the responses to binary outcomes (1 “unfavorable attitude” and 0 “favorable attitude”) the Cronbach alpha value for those four attitudinal statements was 0.664, indicating moderate internal consistency. The inter‐item correlation for these attitudinal statements ranged between 0.207 and 0.612. After unifying the direction of all the statements, a confirmatory factor analysis using principal component analysis extraction, enforcing a single factor, was used to construct the final measure for this group of attitudinal statements. The factor loadings for NegWomanHealth are presented in Table 1 and explain 50% of the variance in the data.

**TABLE 1:** Factor loadings for attitudinal measure NegWomanHealth.

Factor/Attitude	Loadings
FP methods are harmful to the health of the woman	0.775
FP use can affect fertility and prevent women from getting pregnant in the future	0.792
An IUD can move into the abdomen	0.579
Contraceptive pills cause cancer for women	0.664

Abbreviation:FP, family planning.

The remaining four attitudinal statements each reflected a common cultural belief. These variables were not combined in a single index due to the low Cronbach's alpha value for those attitudinal statements, which was 0.233, and the inter‐item correlation ranged between −0.039 and 0.290. The low correlation was expected, as each of those statements focuses on a different construct, which does not necessarily correlate with the others, namely, religious beliefs, family formation obligations, financial readiness, and fertility responsibilities. As such, these variables were examined separately.

#### 2.5.3 Behavioral Outcome Variable

The behavioral outcome variable was *use of a modern FP method at the time of data collection*. Respondents were asked if they (or their spouses) were using *any* FP method at the time of the interview. Those who were using a modern FP method were counted as “users.”

#### 2.5.4 Control Variables

In addition to segregating the analysis by gender, the control variables used in this analysis included respondents’ background characteristics such as age, rural or urban residence, level of education, and number of living children.

### 2.6 Data Analysis

The data were analyzed using SPSS version 26 software. Descriptive statistics were employed to describe the socio‐demographic characteristics, knowledge, attitude, and behavior variables. Chi‐squared test (*χ* ^2^) was employed to determine the association between the respondents’ gender, his/her FP use, and attitudes towards FP. A Student *t*‐test was used to measure gender differences with regard to knowledge and attitude scores, respondent's age, and number of living children. Associations were considered statistically significant at *p* value ≤ 0.05. Finally, two separate logistic regression models were developed to identify the controlled effect of socio‐demographic variables, knowledge, and attitudes on the use of modern FP methods among married male and female respondents, separately.

## 3 Results

### 3.1 Respondent Characteristics

The majority of the sampled factory workers lived in the city of Alexandria; however, this percentage was higher among female than male workers 93% versus 84%, respectively (*p* value = 0.000; Table 2). In terms of age, workers were on average 35 years old with female workers being slightly older than their male counterparts (mean age = 36 and 35 years, respectively, *p* value = 0.000; Table 2). Moreover, female workers in the sample had significantly more children than their male counterparts with approximately 30% of males having 3 or more children compared to 44% of females (*p* value = 0.000; Table 2). Additionally, the average age of their youngest child was significantly lower among male compared to female workers (3.5 and 8.0, respectively, *p* value = 0.000; Table 2). With regards to education, significantly more male than female respondents completed secondary or above secondary education (67% of males compared to 46% of females, *p* value = 0.000; Table 2).

**TABLE 2:** Percentage distribution of surveyed male and female factory workers by selected socio‐demographic characteristics.

	Males (*n* = 406)	Females (*n* = 393)	Total (*n* = 799)
**Residence** ^***^			
Urban	84.2%	93.4%	88.7%
Rural	15.8%	6.6%	11.3%
**Age**			
18–24	2.5%	5.9%	4.1%
25–29	20.2%	13.5%	16.9%
30–34	30.0%	20.4%	25.3%
35–39	25.9%	24.4%	25.2%
40–44	13.1%	23.7%	18.3%
	8.4%	12.2%	10.3%
Mean age (SD)^***^	34.6 (6.4)	36.3 (6.9)	35.5 (6.7)
**Highest level of education completed** ^***^			
No education/reads and writes	8.9%	24.4%	16.5%
Primary or Preparatory	24.1%	29.3%	26.7%
Secondary school/vocational	51.2%	37.9%	44.7%
Above secondary	15.8%	8.4%	12.1%
**No. of living children** ^***^			
0	11.3%	13.7%	12.5%
1–2	59.6%	42.5%	51.2%
3–8	29.1%	43.8%	36.3%
Mean no. children (SD)^***^	1.9 (1.6)	2.3 (1.4)	2.1 (1.3)
**Age of youngest child (total married males with children = 360 and females = 339)**			
Under 1 year	23.6%	5.6%	14.9%
1–2 years	29.2%	12.4%	21.0%
3+ years	47.2%	82.0%	64.1%
Mean age of youngest child (SD)^***^	3.5 (3.8)	8.0 (5.6)	5.7 (5.2)

* *p* value ≤ 0.05.

** *p* value ≤ 0.01.

*** *p* value ≤ 0.00.

### 3.2 Knowledge, Attitudes, and Practices of FP

On average, female factory workers were able to name more modern contraceptive methods than their male counterparts (3.7 vs. 2.8 methods, respectively, *p* value = 0.000; Table 3). Although 50.5% of male respondents were able to mention three to four modern FP methods, this percentage reached 72.8% among their female counterparts (*p* value = 0.000; Table 3).

**TABLE 3:** Percentage distribution of family planning (FP) knowledge among male and female factory workers.

	Males (*n* = 406)	Females (*n* = 393)	Total (*n* = 799)
**Knowledge of modern contraceptives** ^***^			
Doesn't know any	4.2%	0.5%	2.4%
1–2 modern methods	35.9%	10.2%	23.3%
3–4 modern methods	50.5%	72.8%	61.4%
5+ modern methods	9.4%	16.5%	12.9%
Mean no. mentioned (SD)^***^	2.8 (1.3)	3.7 (1.1)	3.2 (1.2)

* *p* value ≤ 0.05.

** *p* value ≤ 0.01.

*** *p* value ≤ 0.00.

Figure 2 shows workers’ agreement/disagreement with each attitudinal statement broken down by respondent's gender. Overall, attitudes among respondents were not highly favorable, with female workers being less favorable compared to males. Least favorable attitudes among both male and female workers were noted with regard to using FP before having the first child, where the percentage of participants who disagreed with the statement “a woman must have at least one child before using an FP method” was 14.5% and 28.9% among female and male workers, respectively (*p* value = 0.000). Moreover, significantly more male than female workers disagreed with the statement “an IUD can move to the abdomen” (49.6% vs. 26.5%, respectively, *p* value = 0.000), and a significantly higher percentage of male than female workers agreed with the statement “newlyweds should postpone pregnancy for 1–2 years after marriage until they are ready” (47.7% and 27.5%, respectively, *p* value = 0.000).

On the other hand, fewer men than women disagreed with the statement “FP is the responsibility of the woman alone” (88.9% vs. 95.9%, respectively, *p* value = 0.001). Moreover, more women than men agreed with the statement “FP use is religiously permissible (not sinful)” (81.2% and 75.6%, respectively, *p* value = 0.014). It is noteworthy that more male respondents answered “don't know” to all the attitudinal statements, except the statement “contraceptive pills can cause cancer,” where the percentage of female respondents who answered “don't know” reached 43% compared to 35% among males (Figure 2).

**FIGURE 3:**
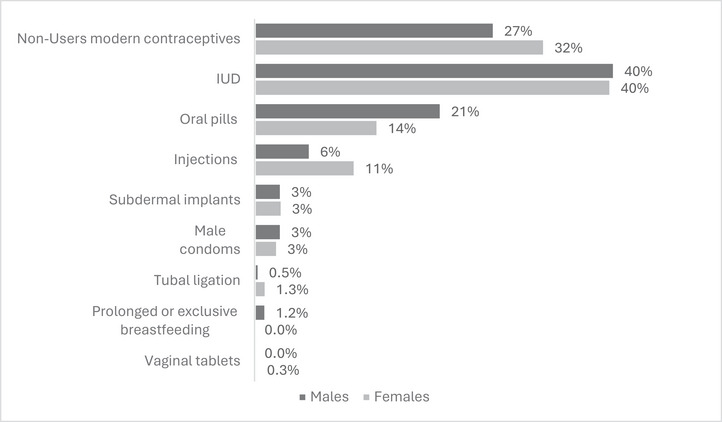
Percentage distribution of current use of FP by type of method and respondent's gender (males = 404, females = 393). Multiple response variables as such responses do not add up to 100%. *Note:* Family planning use refers to their or their spouse use. Two male respondents reported that they did not know whether their spouse was currently using a method, hence excluded in the behavioral variable (current use of FP methods) analysis. As such the total sample of male respondents is 404 when dealing with this behavioural variable. 10 women and 4 men mentioned using more than one method. Ten women and four men mentioned using more than one method.

More male than female workers reported that they (or their spouses) were currently using modern contraceptive methods (73% vs. 68%, respectively, *p* value = 0.041). Respondents were asked to list any FP methods they (or their spouses) were using. The most frequent method was the IUD (reported by nearly 40% of male and female respondents), followed by oral pills (21% and 14%, respectively) and injections (6% and 11%, respectively). The use of other methods (including subdermal implants, male condoms, and vaginal tablets) did not exceed 4% each (Figure 3).

### 3.3 Factors Associated With Use of Modern FP Methods Among Male and Female Factory Workers

Table 4 shows the percentage of current use of modern contraceptives by predictor variables among currently married male and female factory workers. Married male FP users were significantly older than non‐users (35 vs. 33 years, respectively, *p* value = 0.000). Moreover, the percentage of users among males with secondary education or above was higher than among males with lower education levels (76.5% and 66.7%, respectively, *p* value = 0.025). Male users also had more children compared to non‐users (mean = 2.2 vs. 1.1, respectively, *p* value = 0.000). Male workers who lived in rural areas were more likely than their urban counterparts to be using modern FP methods; however, this difference was not statistically significant (79.7% and 72.1%, respectively, *p* value = 0.132).

**TABLE 4:** Percentage distribution of modern family planning (FP) use among male and female workers by selected socio‐demographic characteristics, knowledge, and attitudinal indicators.

Users of modern FP methods	Male workers (*n* = 403)	Female workers (*n* = 393)
**Age**		
Mean age among users (SD)	35.2 (6.1)^**^	36.1 (6.4)
Mean age among non‐users (SD)	32.8 (6.9)^**^	36.7 (7.8)
**Education**		
% use among those with secondary or tertiary level or above	76.5%^*^	70.9%
% use among those with preparatory education or below	66.7%^*^	64.9%
**Number of children**		
Mean number of children among users (SD)	2.2 (1.0)^***^	2.6 (1.2)^***^
Mean number of children among non‐users (SD)	1.1 (1.2)^***^	1.5 (1.5)^***^
**Residence**		
% use among urban residents	72.1%	66.5%
% use among rural residents	79.7%	84.6%
**FP knowledge**		
Mean number of modern methods mentioned among users (SD)	3.0 (1.2)^***^	3.7 (1.0)
Mean number of modern methods mentioned among non‐users (SD)	2.4 (1.4)^***^	3.6 (1.2)
**FP attitudes**		
Mean NegWomenHealth score among users (SD)	43.3 (36.1)^***^	54.7 (34.8)
Mean NegWomenHealth score among non‐users (SD)	58.0 (34.8)^***^	57.6 (32.2)
% use among those who agree that FP is the responsibility of the women alone	68.3%	78.6%
% use among those that disagree (or don't know) that FP is the responsibility of the women alone	74%	67.3%
**Users of modern contraceptives**	73.3%	67.7%

*Note:* One case refused to answer all attitudinal questions, and two cases were unsure whether their spouse was using an FP method and were thus excluded listwise from the regression analysis. Scores are re‐scaled between min 0 and max 100 for percentage scores in this table.

* *p* value ≤ 0.05.

** *p* value ≤ 0.01.

*** *p* value ≤ 0.00.

Male users scored significantly lower on average than non‐users on the negative attitude indicators, namely, “NegWomenHealth” (mean score = 43.3 vs. 58.0, respectively, *p* value = 0.000), that is, non‐users had stronger unfavorable attitudes about the effects of FP on a woman's health. The percentage of male workers who disagreed with the statement “FP was the responsibility of the woman alone” was higher among users than non‐users (74% and 68%, respectively); however, this difference was not statistically significant (*p* value = 0.253). It is noteworthy that the effect of this statement was statistically significant after controlling for the effect of the other independent variables (see Table 5 for the regression model below). Additionally, married males who used modern contraceptives could mention significantly more modern contraceptive methods than non‐users (mean number of mentioned methods = 3.0 vs. 2.4, respectively, *p* value = 0.000).

**TABLE 5:** Factors associated with current use of modern family planning (FP) methods among married male factory workers.

			95% CI for odds	
Male sample = 403	Sig.	Odds	Lower	Upper
Urban residence	0.269	0.653	0.307	1.390
Age^**^	0.002	1.644	1.205	2.244
Age_squared^**^	0.001	0.993	0.989	0.997
Number of children^***^	0.000	2.567	1.879	3.505
Secondary or tertiary education^*^	0.052	1.709	0.995	2.934
Number of modern FP methods mentioned	0.023	1.221	0.972	1.532
NegWomenHealth^**^	0.002	0.655	0.502	0.855
Agree w/FP is the responsibility of the woman alone^*^	0.035	0.394	0.165	0.938
Constant	0.001	0.000		

*Note:* Due to the non‐linear relation between use of modern contraceptives and age in the dataset, observed by plotting age against usage (plot not shown); the variables age and age_squared were entered in the model. One case refused to answer all attitudinal questions, and two cases were unsure whether their spouse was using a FP method, thus excluded listwise from the regression analysis. Nagelkerke *R* square = 0.323. Overall percentage of correct prediction = 82.1%.

* *p* value ≤ 0.05.

** *p* value ≤ 0.01.

*** *p* value ≤ 0.00.

Among female workers, number of living children was the only variable that was found to be significantly associated with modern FP use. The mean number of living children among female workers who were using FP was 2.6 compared to 1.5 among non‐users (*p* value = 0.000).

The logistic regression model for married male factory workers is displayed in Table 5, and the model for married female factory workers is displayed in Table 6. For each additional child that a married male factory worker has, while holding all other variables constant, the likelihood of modern FP use increases by nearly 2.6 times. Age significantly increases the likelihood of using modern contraceptives; however, the effect of age decreases as the respondent gets older. With regard to attitudes, the likelihood of modern FP use decreased by approximately 35% among those with unfavorable attitudes (i.e., higher “NegWomenHealth” score) and decreased by approximately 61% among those who believed that FP is the woman's responsibility alone. Additionally, basic knowledge of FP methods was associated with increased likelihood of use, with the likelihood of use increasing by 1.2 times with every additional modern contraceptive mentioned by male respondents.

**TABLE 6:** Factors associated with current use of modern family planning (FP) methods among married female factory workers.

			95% CI for Exp(*B*)	
Female sample = 393	Sig.	Exp(*B*)	Lower	Upper
Urban residence	0.417	0.611	0.186	2.009
Age	0.064	1.357	0.983	1.874
Age_squared	0.026	0.995	0.990	0.999
Number of children^***^	0.000	1.976	1.607	2.431
Secondary or tertiary education	0.238	1.338	0.825	2.173
Number of modern FP methods mentioned	0.302	1.127	0.898	1.413
NegWomenHealth	0.588	0.935	0.732	1.194
Agree w/FP is the responsibility of the woman alone	0.312	2.006	0.520	7.733
Constant	0.090	0.008		

*Note:* Nagelkerke *R* square = 0.241. Overall percentage of correct prediction = 76.1%.

* *p* value ≤ 0.05.

** *p* value ≤ 0.01.

*** *p* value ≤ 0.00.

On the other hand, among married female factory workers, for each additional child, the likelihood of using modern FP contraceptives increases by over two‐fold (2.4 times), while holding all other variables constant. None of the other variables in the model was significantly associated with the use of modern FP (Table 6).

## 4 Discussion

The present study examined FP knowledge, attitudes, and practices among male and female garment factory workers, as well as factors associated with modern contraceptive use among each of the two groups.

Results of our study showed a higher level of knowledge of FP methods among female than male workers, where the former knew an average of 3.7 methods compared to 2.8 methods for their male counterparts. Those results are in line with evidence from other countries, such as Cambodia, Ghana, Pakistan, Indonesia, and India, which shows married and unmarried women to be more knowledgeable about FP methods than their male counterparts [18]. Higher knowledge among women may be because awareness campaigns, in most countries, have traditionally targeted women, assuming that men are not interested in learning about FP. Research from the MENA region, India, and Pakistan confirms that FP is perceived as a women's business, and hence women may be more knowledgeable of FP than their male counterparts [19, 20, 21].

However, female factory workers in this study expressed more health concerns and misconceptions about FP methods compared to their male counterparts. This may be because women are more likely than men to discuss FP with their family and friends, hence sharing experiences and concerns, including misinformation [22, 23].

Most men and women in this study believed that a woman must have at least one child before using FP. However, significantly more women than men expressed this belief (83% of women compared to 67% of men). This could be a result of women's concerns and fears about the side effects of contraception. This finding also reflects inequitable gender norms whereby women are expected to demonstrate their ability to conceive before using FP. Similarly, studies in Bangladesh and India show that even though married women may express a desire for using FP, they often feel pressure to prove their fertility and thus are likely to postpone using contraception or discontinue using one [2, 24].

Married men, on the other hand, appeared less determined in their attitudes about the potential effects of FP methods on a woman's health. This finding may be due to the fact that men are less knowledgeable about FP, are less likely to talk about it with their peers, and hence do not have preformed ideas about its pros and cons. This finding is consistent with findings from South Africa, which showed men to be ambivalent about FP despite their acknowledgment of its benefits [25, 26].

Interestingly, more men in this study had misconceptions about the religious standpoint regarding FP or were unsure about it. Moreover, more men believed that FP is a woman's responsibility. Those results are in line with those of studies from India and several MENA countries, which showed that men considered FP as a woman's responsibility [19, 21, 27].

Factors that were associated with the use of modern FP methods among male workers (or their spouses) were the number of living children, a worker's age, and his attitudes towards FP. The more children a worker has, the more likely he (or his spouse) is to use modern contraception. Negative attitudes about potential effects of contraception on a woman's health and a belief that FP is a woman's responsibility were both negatively associated with reporting use of a modern FP method among men. Moreover, knowledge of more FP methods was associated with increased use of modern contraception among men (or their spouses). The above findings are similar to those reported by Bueno Cababaro et al., which showed that in the Philippines, men with good knowledge, more children, and more favorable attitudes about FP were likely to be using FP [28].

On the other hand, the only factor that was associated with modern FP use among female workers was the number of living children a woman had. In other words, among married female factory workers, a woman is likely to use FP only when she has had “enough children,” regardless of her attitudes or knowledge about FP. This finding is consistent with research from Ethiopia, which showed that higher knowledge of contraceptives was not associated with increased use [29]. Likewise, a study from India showed that favorable attitudes about FP among married women were not predictive of FP use [30].

The above findings suggest that contextual factors related to gender norms and power dynamics within the household may play a greater role in determining women's use of FP than their knowledge and attitudes. A study by Jejeebhoy and colleagues in India (2014) found that women's participation in marriage‐related decision‐making was positively associated with use of FP to delay first pregnancy, while experiencing pressure from family or in‐laws to prove their fertility was associated with a lower likelihood of FP use to delay first pregnancy. Earlier research from the US, using an expanded version of the Health Belief Model (HBM) in the prediction of condom use, showed that high levels of HIV/AIDS risk knowledge were not significantly correlated with condom use. The findings suggest a need to consider additional (e.g., sociocultural) factors associated with African American sexual decision‐making and condom use to develop applicable conceptual models and HIV/AIDS prevention approaches [31]. Our findings highlight the need for gender‐specific interventions that address the distinctive FP needs of male and female factory workers. Messages for both male and female factory workers should emphasize the benefits of birth spacing and rectify misconceptions about side effects of FP methods, especially those related to potential effects on future fertility. Messages to male workers should clarify Islam/Christianity's position vis‐à‐vis FP and emphasize male responsibility in FP, whereas female workers may need life skills training to enhance their negotiation skills, self‐efficacy, and their awareness of their reproductive rights [32].

On‐site provision of FP information and services to factory workers could help in addressing their unmet FP needs and rectifying misconceptions about FP. Research from Cambodia, Vietnam, and Bangladesh has shown that female factory workers are not able to access FP services because they have no time after work to seek those services [8, 9, 23]. FP information could be offered through the factory nurse, trained male and female peer educators, social and behavior change materials, as well as social media [7, 10]. Last but not least, gender stereotypes and structural factors that limit women's decision‐making power should be addressed.

### 4.1 Study Limitations

The present study was conducted in 14 factories, the owners of which agreed to participate in a project to address workers’ FP/RH needs. Those factories may not be representative of other garment factories in the two industrial zones in terms of worker socio‐demographic characteristics, FP knowledge, attitudes, and practices. Moreover, factory workers who participated in the survey were not selected through randomization. Respondents were the ones who were released by their line supervisors; hence, they may not be representative of male and female workers who were employed in those factories at the time of data collection. However, it is worth noting that factory managers and supervisors in both industrial zones were informed at the onset of the project that their factories would be receiving an intervention to improve workers’ access to FP/RH information and services and that this baseline was in no way an evaluation of those factories. This should minimize selection bias, as supervisors would see no benefit in releasing workers with specific socio‐demographic characteristics or those with more or less favorable attitudes about FP/RH. Our measure of FP knowledge was based on the FP methods that were listed by respondents and did not solicit information on respondents’ in‐depth knowledge of FP methods (e.g., mode of action and potential side effects). This may explain the finding that FP knowledge among female workers was not found to be associated with FP use.

Moreover, reports of FP use among male workers may not be accurate, as husbands may not be fully aware of their wives’ FP practices, that is, whether or not their wife was currently using a method or the type of method she was using. Finally, the survey did not ask female respondents about their spouses’ FP attitudes or fertility desires, although husbands’ preferences may be one of the stronger predictors of FP use, especially in resource‐poor settings [20, 21, 22, 27].

## 5 Conclusions and Recommendations

Although previous research has focused on the FP needs of female factory workers and did not distinguish between the information needs of males and females, to our knowledge, this is the first study to provide gender‐disaggregated data on garment factory workers’ knowledge, attitudes, and practices related to FP and to identify predictors of modern use of contraception among each of those two groups. The study revealed a clear influence of inequitable gender norms and dynamics on male and female factory workers’ FP knowledge, attitudes, and practices. Findings of this study suggest that this population of factory workers still sees FP as a woman's business, as female workers were more informed about FP methods but more concerned about the potential effect of contraception on their subsequent fertility. Increased knowledge of FP and more favorable attitudes were associated with FP use among male workers (and their spouses), but for female workers, the only predictor was a higher number of children. This indicates that, regardless of their beliefs or preferences, female factory workers may not use modern contraception until they have had “enough” children and fulfilled the role prescribed for them by society. Several recommendations are proposed to address male and female workers’ distinctive FP needs, namely, on‐site provision of FP information and services at the workplace and addressing gender norms that limit women's decision‐making power and hinder male involvement in FP. Future research that examines FP use among women should integrate data on husband attitudes, as male opposition or support is a critical determinant of women's contraceptive decisions in many low‐resource settings.

## Author Contributions

Both Noha el Khorazaty and Nahla Abdel‐Tawab developed the research's theoretical formalism and wrote the final version of the manuscript. Noha el Khorazaty performed the statistical analysis and initial draft of the manuscript. Nahla Abdel‐Tawab led the design and implementation of the project and the final draft of the manuscript.

## Funding

The project “Offering Family Planning Services in Factory Settings in Egypt” is implemented by Population Council/Egypt with funds from the Embassy of the Kingdom of the Netherlands (Contract/Award no.: 4000005062).

## Ethics Statement

Ethical study approval was obtained from the Population Council Institutional Review Board (New York, NY, USA), and each factory management approval was obtained to conduct the overall project in January 2022.

## Consent

Additionally, informed consent was obtained from all respondents before each interview. All participants provided verbal informed consent prior to participation, and procedures to maximize the safety of participants are detailed in our study protocol.

## Conflicts of Interest

The authors declare no conflicts of interest.

## Data Availability

The datasets used and analyzed in the current study are available from the corresponding author upon reasonable request.
